# CXCL10 Signaling Contributes to the Pathogenesis of Arthritogenic Alphaviruses

**DOI:** 10.3390/v12111252

**Published:** 2020-11-02

**Authors:** Tao Lin, Tingting Geng, Andrew G. Harrison, Duomeng Yang, Anthony T. Vella, Erol Fikrig, Penghua Wang

**Affiliations:** 1 Department of Immunology, School of Medicine, University of Connecticut Health Center, Farmington, CT 06030, USA; tlin@uchc.edu (T.L.); geng@uchc.edu (T.G.); aharrison@uchc.edu (A.G.H.); dyang@uchc.edu (D.Y.); vella@uchc.edu (A.T.V.); 2 Section of Infectious Diseases, School of Medicine, Yale University, New Haven, CT 06520, USA; erol.fikrig@yale.edu

**Keywords:** CXCL10, alphavirus, Chikungunya virus, O’nyong nyong virus, viral arthritis

## Abstract

Emerging and re-emerging arthritogenic alphaviruses, such as Chikungunya virus (CHIKV) and O’nyong nyong virus, cause acute and chronic crippling arthralgia associated with inflammatory immune responses. Approximately 50% of CHIKV-infected patients suffer from rheumatic manifestations that last 6 months to years. However, the physiological functions of individual immune signaling pathways in the pathogenesis of alphaviral arthritis remain poorly understood. Here, we report that a deficiency in CXCL10, which is a chemoattractant for monocytes/macrophages/T cells, led to the same viremia as wild-type animals, but fewer immune infiltrates and lower viral loads in footpads at the peak of arthritic disease (6–8 days post infection). Macrophages constituted the largest immune cell population in footpads following infection, and were significantly reduced in *Cxcl10*^−/−^ mice. The viral RNA loads in neutrophils and macrophages were reduced in *Cxcl10*^−/−^ compared to wild-type mice. In summary, our results demonstrate that CXCL10 signaling promotes the pathogenesis of alphaviral disease and suggest that CXCL10 may be a therapeutic target for mitigating alphaviral arthritis.

## 1. Introduction

Alphaviruses are a genus of single-stranded, positive-sense RNA viruses within the *Togaviridae* family. These viruses are mainly transmitted by mosquitoes and pose a public health threat worldwide, particularly in tropical/subtropical regions. Many alphaviruses are arthritogenic, including Chikungunya (CHIKV), O’nyong-nyong (ONNV), and Ross River viruses (RRV), etc. CHIKV is the causative agent of acute and chronic crippling arthralgia that was initially identified in Tanzania in 1952 [1]. Since then, several major epidemics have been recorded on the Indian Ocean islands, India, Southeast Asia, which resulted in over 6 million cases [2]. In late 2013, CHIKV emerged on the Caribbean islands, and has now spread to more than 50 countries across Central and South America, including autochthonous infections in the United States, and caused over 2.5 million infections (Sources: Pan America Health Organization). Approximately 50% of CHIKV-infected patients suffer from rheumatic manifestations that last 6 months to years, with ~5% of the victims having rheumatoid arthritis-like illnesses [3,4].

However, no licensed specific antiviral therapeutics or vaccines are available worldwide. This is partly due to the lack of an in-depth understanding of the pathogenic mechanisms of CHIKV in immune-competent animal models. It has become clear that innate immunity, in particular the type I IFN system, is one of the most important early antiviral mechanisms in response to alphaviruses. During the acute phase of infection in humans (~two weeks), CHIKV infects many organs and cell types [2], inducing apoptosis and direct tissue damage [5,6,7]. The acute phase is also characteristic of robust innate immune responses, including high levels of type I IFNs, proinflammatory cytokines/chemokines, and growth factors [5,8,9,10,11,12]. Immune cell infiltration is a hallmark of acute CHIKV infection, primarily including macrophages and monocytes, but also neutrophils, dendritic cells, NK cells, and lymphocytes [2]. In the chronic phase, CHIKV arthritis may progress without active viral replication, typified by an elevated expression of cytokines and immune cell infiltration [2,13]. In particular, the human arthritic disease severity is associated with a high level of serum chemoattractants for monocytes/macrophages/T cells, CXCL10, and CXCL9 [14]. In mice, CHIKV infection leads to a low viremia usually lasting 5–7 days, which is limited by type I IFNs [11,15,16,17] and is subsequently cleared by virus-specific antibody responses [18,19,20,21,22,23,24,25]. When injected directly into a mouse foot pad, CHIKV elicits overt arthritic symptoms, including the first peak of foot swelling characteristic of edema occurring 2–3 days post infection and a second peak at 6–8 days post infection [26], with extensive infiltration of immune cells into the infected feet [27,28,29,30,31].

Various classes of active compounds have been reported to inhibit CHIKV replication; however, the in vivo efficacy and safety of these candidates has not been evaluated in animal models [32]. A live attenuated CHIKV virus vaccine was efficacious in 85% of vaccinated people after one year in a phase II trial, but 8% of vaccinated people reported transient joint pain, representing a potential safety concern and enforcing the need for more research on therapies [33]. At present, there is still a great need for safe and effective prophylactics and therapeutics. Using mouse models of viral infection and innate immunity, here, we report that CXCL10 signaling promotes the pathogenesis of alphaviral disease. *Cxcl10*^−/−^ mice had the same viremia as wild-type animals, but fewer immune infiltrates and lower viral loads in footpads at the peak of arthritic disease (days 6–8 post infection). Macrophages constituted the largest immune cell population in footpads following infection, and were significantly reduced in *Cxcl10*^−/−^ mice. The viral RNA loads in neutrophils and macrophages were also reduced in Cxcl10^−/−^ compared to wild-type mice.

## 2. Materials and Methods

### 2.1. Mice

All the mice used in this study were purchased and bred in our state-of-the-art animal facility. Wild-type C57BL/6J (JAX Stock #: 000664) and *Cxcl10*^−/−^ (JAX Stock #: 000687 on C57BL/6J background) mice were obtained from the Jackson Laboratory. For each experiment, both sex (both genders)- and age (range of 6–12 weeks)-matched WT/mutant mice were used. Mouse experiments were approved and performed according to the guidelines of the Institutional Animal Care and Use Committee at the University of Connecticut and Yale University.

### 2.2. Cells and Viruses

Vero cells (monkey kidney epithelial cells, Cat. # CCL-81) were purchased from ATCC (Manassas, VA, USA). The cells were grown at 37 °C and 5% CO_2_ in complete Dulbecco’s modified Eagle medium (DMEM) medium: DMEM (Corning) supplemented with 10% fetal bovine serum (FBS) (Gibco) and 1% penicillin-streptomycin (P/S; Corning, Glendale, Arizona, US). The CHIKV French La Reunion strain LR2006-OPY1 was a kind gift of The Connecticut Agricultural Experiment Station located in New Haven, CT, USA. The ONNV non-recombinant strain was provided by the World Reference Center for Emerging Viruses and Arboviruses (WRCEVA) at the University of Texas Medical Branch. Both viruses were propagated in Vero cells.

### 2.3. Plaque Forming Assay

The quantification of infectious viral particles in cell culture supernatants/mouse tissue homogenates/mouse sera was performed on a Vero cell monolayer in a 6-well plate, following an established protocol [34]. A series of 10-fold dilutions of viral samples were prepared in DMEM without fetal bovine serum. In a 6-well plate, 500 µL of diluted samples were added to a Vero monolayer. The plate was incubated at 37 °C and 5% CO_2_ for 2 h. The inoculum was then removed and replaced with 2 mL of complete DMEM medium with 1% SeaPlaque agarose (Cat# 50100, Lonza, Morristown, NJ, US). The plate was incubated at 37 °C and 5% CO_2_ for 3 days, and plaques were visualized by a Neutral Red exclusion assay. Viable cells took up neutral red, while dead cells excluded it and thus formed a circular white spot.

### 2.4. Mouse Infection and Disease Monitoring

Age- and sex-matched mice were inoculated subcutaneously in the hind footpad with 3 × 10^5^ plaque forming units (PFUs) of CHIKV/ONNV. Mice were then monitored for clinical signs of disease. Footpad swelling was measured using a precision digital caliper. Foot swelling as an indicator of local inflammation was recorded over a period of 8 days after infection. The thickness and width of the perimetatarsal area of the hind feet were measured using a precision metric caliper in a blinded fashion. The foot dimension was calculated as width × thickness, and the results were expressed as the fold increase in the foot dimension after infection compared to before infection (day 0 baseline).

### 2.5. Histology Studies

Mice were sacrificed and feet were removed and fixed with 4% paraformaldehyde. Tissues were embedded in paraffin and processed to obtain 5 μm sections. Tissues were stained with hematoxylin and eosin. Arthritic disease was arbitrarily scored from 1 to 5, with 5 representing the worst, based on the exudation of fibrin and inflammatory cells into the joints, alteration in the thickness of tendons or ligament sheaths, and hypertrophy and hyperplasia of the synovium [35]. Slides were imaged using an Accu-Scope EXI-310 model inverted microscope with Infinity Capture software.

### 2.6. Treatment with Dimethyl Sulfoxide (Vehicle) and Atorvastatin In Vivo

Atorvastatin was dissolved in dimethyl sulfoxide. Mice were injected intraperitoneally with dimethyl sulfoxide (vehicle) or atorvastatin (25 mg/kg/day) (Cat#1044516, Sigma-Aldrich, St. Louis, MO, US) from Day 3 through 8 post CHIKV infection (p.i.) on a daily basis.

### 2.7. Flow Cytometry and Florescence Activated Cell Sorting

Mice were euthanized, and footpads and ankles were harvested at 0, 2, 4, and 6 days post infection (dpi). The footpads were skinned and put into 4 mL of digestion medium with 20 mg/mL collagenase IV (Sigma-Aldrich, St. Louis, MO, US), 5 U/mL dispase (Stemcell, Cambridge, MA, US), and 50 mg/mL DNase I mix (Qiagen, Germantown, MD, US) in complete RPMI1640 medium. The tissues were harvested and incubated in digestion medium on a shaker at 37 °C for 4 h. The mixture was transferred to a 40 μm cell strainer sitting on a collection tube. In total, 5 mL of complete RPMI medium was added to the cell strainer. Using a circular motion, the digested tissues were ground into the medium against the cell strainer to release the maximum number of cells. Cells were then centrifuged at 500× *g* for 5 min. The supernatant was discarded, and red blood cells were lysed using 0.2% sodium chloride. Cells were washed once in complete RPMI medium, and re-suspended in 10 mL of complete RPMI medium in a 15 mL tube. In total, 10 mL of 35% *v*/*v* Percoll/RPMI medium was carefully added to the cell suspension. The tube was spun for 20 min at 1200× *g*. The pellet was re-suspended and washed with complete RPMI medium once.

The isolated cells were then stained for 30 min at 4 °C with the following antibodies (Biolegend, San Diego, CA, US): APC-Fire 750-anti CD11b (Cat. # 101261); Alexa Flour 700-anti Ly-6G (Cat. # 127621); Brilliant Violet 421-anti CD11c (Cat. # 117343); PerCP-Cy5.5-anti MHC II (Cat. # 107625); PE-anti Tetherin (PCDA1) (Cat. # 12703); Brilliant Violet 510-anti F4/80 (Cat. # 123135); APC-anti CD68 (Cat. # 137007); PE-Dazzle 594-anti CD3 epsilon (Cat. # 100347); Brilliant Violet 711-anti CD4 (Cat. # 100557); Brilliant Violet 570-anti CD8a (Cat. # 100739); FITC-anti CD25 (Cat. # 102005); Zombie UV (Cat. # 423107); PE-Cy7-anti CD45 (Cat. # 103113); and TruStain FcX-anti CD16/32 (Cat. # 101319). After staining and washing, the cells were fixed with 4% PFA and analyzed by fluorescence activated cell sorting (FACS).

Flow cytometry was later performed on a Becton-Dickinson FACS ARIA II, CyAn advanced digital processor (ADP) and analyzed using FlowJo software. Neutrophils were classified as CD11b+ Ly6G+, macrophages were classified as CD11b+ F4/80+, DC cells were classified as CD11c+ MHC II+, and pDC cells were classified as CD11c+ PCDA1+.

### 2.8. Real-Time Quantitative RT-PCR

RNA was isolated from blood samples and footpad tissues using an RNAasy mini-prep kit (Invitrogen, Carlsbad, CA, US). For paraformaldehyde-fixed and sorted cells, RNA was isolated using the RNeasy FFPE Kit (Qiagen, Germantown, MD, US). Isolated RNA was resuspended in RNAse/DNAse free H_2_O (Invitrogen) and stored at 4 °C overnight or −80 °C. RT was performed on a Bio-Rad CFX machine using the RNA RT Kit (Takara, Mountain View, CA, US) with a 10 μL total reaction volume per well containing 3 μL of RNA samples. Quantitative PCR (qPCR) was performed with gene-specific primers and SYBR Green. The primers applied for CHIKV were the forward primer (5′-GCGAATTCGGCGCAGCACCAAGGACAACTTCA-3′) and reverse primer (5′-AATGCGGCCGCCTAGCAGCATATTAGGCTAAGCAGG-3′). The primers employed for ONNV were the forward primer (5′-GCAGGGAGGCCAGGACAGT-3)’ and reverse primer (5′-GCCCCTTTTTCYTTGAGCCAGTA-3′). The housekeeping gene control used was beta actin (Actb). The following PCR cycling program was used: 10 min at 95°, and 40 cycles of 15 s at 95° and 1 min at 60 °C. The results were calculated using the -∆∆Ct method.

### 2.9. Statistical Analysis

All data were analyzed with GraphPad Prism software. For viral RNA analysis, immune cell analysis, cytokine and chemokine analysis, and footpad swelling, data were analyzed by the nonparametric Mann–Whitney test, two-tailed Student’s *t* test, or multiple *t*-tests, depending on the data distribution and number of comparison groups. *p* values of less than 0.05 were considered statistically significant.

## 3. Results

### 3.1. CXCL10 Signaling Contributes to Alphavirus Pathogenesis

**Figure 1:**
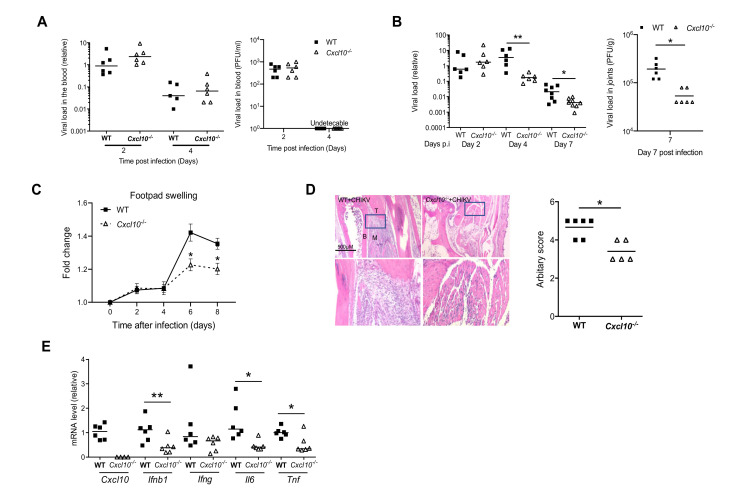
CXCL10 signaling facilitates Chikungunya virus (CHIKV) pathogenesis in mouse feet. Age- and sex-matched C57BL/6 (wild-type (WT)) and *Cxcl10*-deficient (*Cxcl10*^−/−^) mice were infected with CHIKV. (**A**) Quantification of viremia by qPCR in whole blood cells (left panel) and a plaque assay (PFU/mL serum) (right panel) of WT and *Cxcl10*^−/−^ mice. (**B**) Quantification of viral loads by qPCR (left panel) and a plaque forming assay (PFU/g tissue, day 7) at various days after infection. (**C**) Fold changes in the footpad dimensions of CHIKV-infected mice (Days 2, 4, 6, and 8) in comparison to the uninfected mice (Day 0) (*n* = 8 per genotype). Error bar: mean ± s.e.m. *, *p* < 0.05, non-parametric Mann–Whitney test. (**D**) Representative hematoxylin and eosin staining (H&E) micrographs and arbitrary scores of ankle joint inflammation and damage using a scale of 1 to 5, with 5 representing the worst disease at 7 days after CHIKV infection. N = 5–6/genotype. Magnifications 40×. B: bone, T: tendon, and M: muscle. Boxed areas indicate the regions with infiltration and tissue damage. Magnification: 200×. (**E**) qPCR quantification of cytokines in ankle joints at day 8 post infection (p.i.). Each dot = one mouse. The horizontal line in each column = the median. * *p* < 0.05; ** *p* < 0.01 ((non-parametric Mann–Whitney test for (**B**,**D**) and two-tailed Student’s *t*-test for (**E**)).

In humans, Chikungunya arthritis severity is associated with a high level of serum chemoattractants for monocytes/macrophages/T cells, referred to as CXCL10 [14]. To investigate the physiological role of CXCL10 in alphavirus pathogenesis, we injected CHIKV directly into the footpads of both wild-type (WT) and Cxcl10 knockout (*Cxcl10*^−/−^) mice. The results show that the viremia of *Cxcl10*^−/−^ mice were comparable to those in WT mice at days 2 and 4 post infection (p.i.) (Figure 1A), suggesting that CXCL10 is dispensable for controlling the systemic dissemination of CHIKV. The viral loads in the infected ankle joints of WT and *Cxcl10*^−/−^ mice were the same at day 2 p.i. and increased modestly in WT at day 4 p.i., while dropped significantly and rapidly in *Cxcl10*^−/−^ mice from days 4 through 7 (Figure 1B), suggesting that CXCL10 signaling promotes viral persistence. Intriguingly, histopathological analyses conducted by foot swelling monitoring and H&E staining confirmed a moderate decrease in immune cell numbers in the muscles and joints of *Cxcl10*^−/−^ compared to WT mice (Figure 1C,D). Consistently, the mRNA expression of *Ifnb1* and inflammatory cytokines (*Il6*, *Tnfa*) was reduced in *Cxcl10*^−/−^ joints at day 7 p.i. compared to WT (Figure 1E).

**Figure 2:**
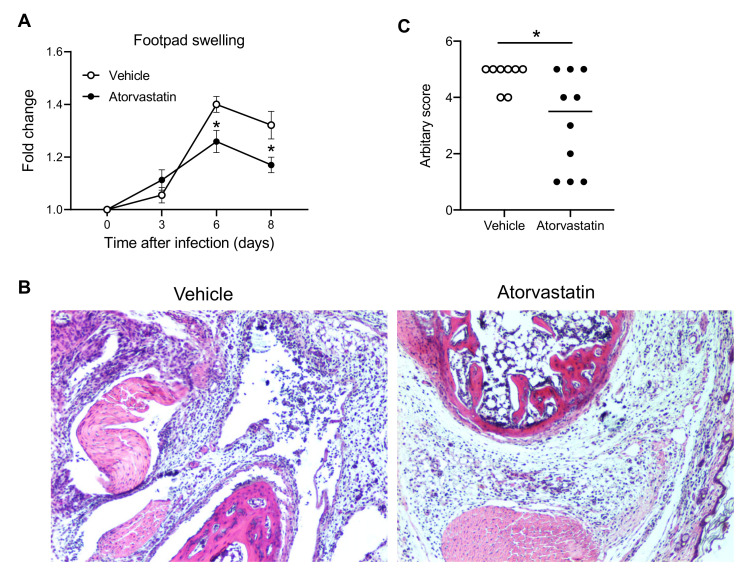
Atorvastain suppresses CHIKV arthritis. Mice were injected intraperitoneally with dimethyl sulfoxide (vehicle) or atorvastatin (25 mg/kg/day) from Day 3 through 8 post CHIKV infection (p.i.) on a daily basis. (**A**) Fold changes in the footpad dimensions of infected mice (Days 3, 6, and 8) in comparison to the uninfected mice (Day 0) (*n* = 8 for vehicle and 10 for atorvastatin). Error bar: mean ± s.e.m. (**B**) Representative H&E micrographs (100×) and (**C**) arbitrary score of H&E on Day 8 p.i. The horizontal line in (**C**) is the median of the results. * *p* < 0.05, two-tailed Student’s *t*-test for (**A**,**C**). Each symbol represents one mouse.

We then used a clinically approved, cholesterol-lowering drug, called atorvastatin, which has recently been demonstrated to effectively inhibit CXCL10 expression in both mice and humans [36,37]. We treated mice with dimethyl sulfoxide (vehicle) or atorvastatin from day 3–8 p.i. [36] during the acute phase of CHIKV infection. Indeed, atorvastatin treatment alleviated CHIKV-induced inflammation and disease (Figure 2), suggesting that CXCL10 could be a potential drug target for treating CHIKV arthritis.

**Figure 3:**
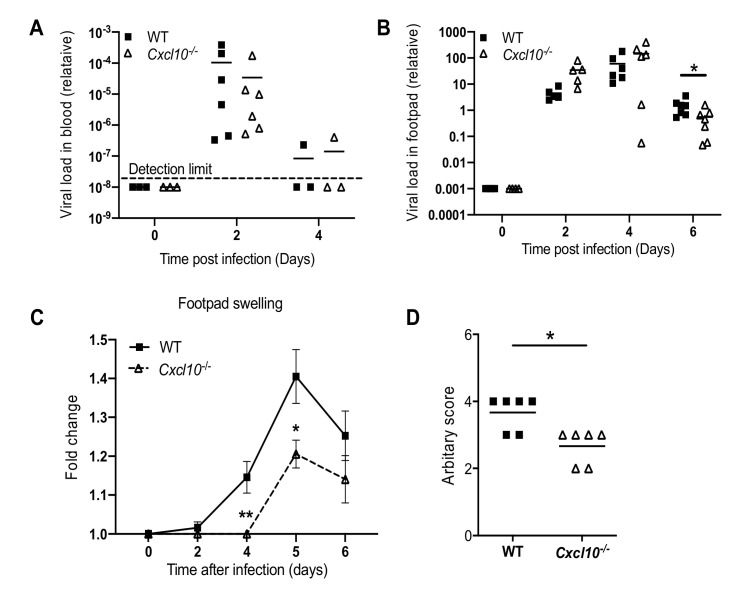
CXCL10 signaling facilitates O’nyong-nyong virus (ONNV_ pathogenesis in mouse feet. Age- and sex-matched C57BL/6 (WT) and *Cxcl10*-deficient (*Cxcl10*^−/−^) mice were infected with ONNV. (**A**) qPCR quantification of ONNV loads in (**A**) whole blood cells and (**B**) the footpads at various days after infection. (**C**) Fold changes in the footpad dimensions of ONNV-infected mice (Days 2, 4, 5, and 6) in comparison to the uninfected mice (Day 0) (*n* = 7 per genotype). Error bar: mean ± s.e.m. * *p* < 0.05, ** *p* < 0.01, two-tailed Student’s t-test. (**D**) Arbitrary scores of ankle joint inflammation and damage using a scale of 1 to 5, with 5 representing the worst disease at 6 days after ONNV infection. N = 6/genotype. Each dot = one mouse. The horizontal line in each column = the median. * *p* < 0.05 (non-parametric Mann-Whitney test for (**B**,**D**)).

We next investigated whether this phenomenon is applicable to other arthritogenic viruses. Considering this, we chose O’nyong-nyong (ONNV), which, together with CHIKV, is a member of the Semliki Forest antigenic complex of the *Alphavirus* genus. Consistent with the results from our CHIKV studies, ONNV viremia were not influenced by *Cxcl10* deficiency (Figure 3A), while the viral loads in the infected feet were lower in *Cxcl10*^−/−^ than those in WT mice at day 6 p.i. (Figure 3B). Fold changes in the footpad dimensions and histopathological analyses (arbitrary score) by hematoxylin and eosin staining (H&E) confirmed a moderate decrease in immune cell infiltration into the muscles and joints of *Cxcl10*^−/−^ compared to WT mice (Figure 3C,D). These data suggest that CXCL10 signaling promotes alphaviral persistence and immune cell infiltration into mouse feet.

### 3.2. CXCL10 Signaling Promotes Macrophage Recruitment to Infected Feet

**Figure 4:**
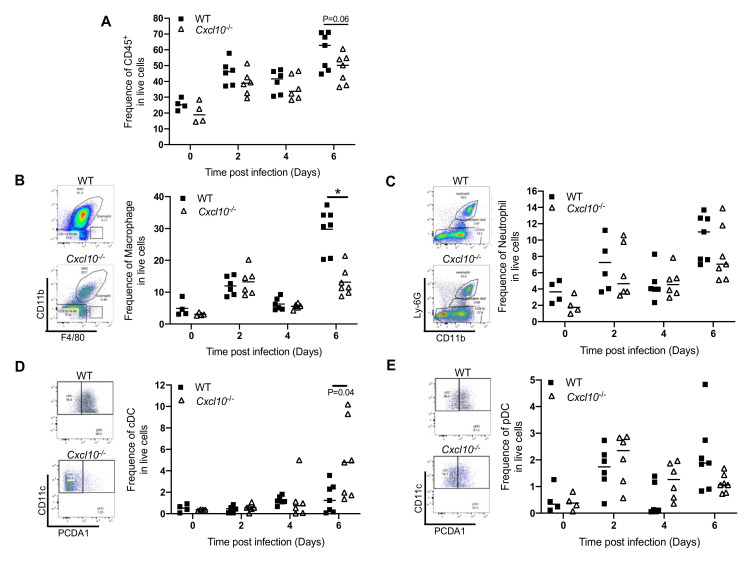
CXCL10 signaling facilitates macrophage infiltration into mouse feet. Age- and sex-matchedC57BL/6 (WT) and *Cxcl10*-deficient (*Cxcl10*^−/−^) mice were infected with ONNV. Different immune cells were quantitated by fluorescence activated cell sorting (FACS). The frequencies of (**A**) the total number of CD45^+^ immune cells, (**B**) CD11b^+^ F4/80^+^ macrophages, (**C**) CD11b^+^ Ly-6G^+^ neutrophils, (**D**) CD11c^+^ MHCII^+^ conventional dendritic cells (cDC), and (**E**) CD11c^+^ PCDA1^+^ plasmacytoid dendritic cells (pDC). Each dot = one mouse. The horizontal line in each column = the median. * *p* < 0.05 (non-parametric Mann-Whitney *t* test).

Since CXCL10 is a chemoattractant for monocytes/macrophages, we analyzed the immune infiltrates in the infected feet by fluorescence activated cell sorting (FACS), in order to identify and quantitate individual cell populations. In WT mice, the total number of CD45^+^ cells increased modestly at day 2 p.i., decreased slightly at day 4 p.i., and then elevated again at day 6 p.i. There was a modest decrease in CD45^+^ cells in *Cxcl10*^−/−^ at day 6 p.i. compared to WT (*p* = 0.06) (Figure 4A). Macrophages were recruited rapidly, as early as day 2 p.i., and were the largest immune population at all censored time points. Intriguingly, these cells were significantly fewer in number in *Cxcl10*^−/−^ than WT mice at day 6 p.i. (Figure 4B). Neutrophils constituted the second largest immune population and infiltrated into the infected feet similarly between WT and *Cxcl10*^−/−^ mice in terms of quantities and kinetics (Figure 4C). Compared to those in the uninfected mice (day 0), the numbers of conventional dendritic cells (cDC) were only significantly increased by day 6 p.i. and higher in *Cxcl10*^−/−^ than WT mice (Figure 4D). Plasmacytoid dendritic cells (pDC) were also recruited to the infected feet as early as day 2 p.i. and their numbers were the same in both genotypes (Figure 4E).

### 3.3. CXCL10 Signaling Promotes Alphavirus Persistence in Infiltrating Neutrophils and Macrophages

**Figure 5:**
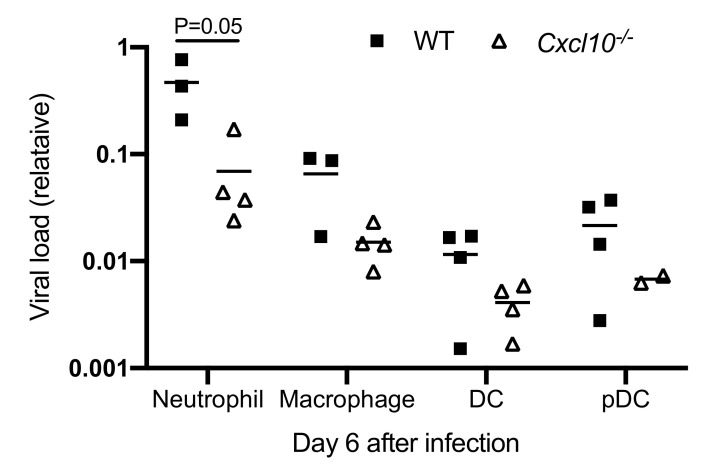
Viral RNA loads are reduced in *Cxcl10*^−/−^ macrophages and neutrophils. Age- and sex-matched C57BL/6 (WT) and Cxcl10-deficient (*Cxcl10*^−/−^) mice were infected with ONNV. Immune cells were sorted by FACS. ONNV RNA in the sorted immune cells was quantitated by RT-PCR. Each dot = one mouse. The horizontal line in each column = the median. *p* values were calculated with the Mann-Whitney test.

The abovementioned data show that macrophages and neutrophils are the primary infiltrating cells in the infected mouse feet, and interestingly, the former has been demonstrated to likely be a source of CHIKV persistence in nonhuman primates [38]. We then examined ONNV RNA loads in each cell population after FACS. We were able to extract RNA from paraformaldehyde-fixed cells using a specialized RNA kit and quantitated viral RNA by qRT-PCR. Among all immune cells, neutrophils contained the highest viral load, which was remarkably reduced in *Cxcl10*^−/−^ mice (*p* = 0.05) compared to WT mice (Figure 5). Macrophages had the second highest viral load, which was also decreased in *Cxcl10*^−/−^ mice. The viral loads in cDC and pDC, though at a much lower level than neutrophils/macrophages, tended to be lower in *Cxcl10*^−/−^ mice (Figure 5). These data suggest that, during the acute phase of infection, neutrophils and macrophages are likely an important source of alphaviral replication in infected tissues, and CXCL10 signaling promotes alphavirus persistence in infiltrating neutrophils and macrophages in the foot.

## 4. Discussion

CXCL10 is a chemoattractant for monocytes/macrophages, T cells, NKs, and DCs, and can also promote T cell adhesion to endothelial cells, antitumor activity, and the inhibition of bone marrow colony formation and angiogenesis [39,40,41]. Intriguingly, a high level of serum CXCL10 is associated with severe arthritic disease in humans [14]. CXCL10 is secreted by several cell types, including monocytes, endothelial cells, and fibroblasts. Its expression is increased in many kinds of chronic inflammatory arthritis, especially in rheumatoid arthritis (RA). It is thus plausible that, during CHIKV infection, CXCL10 plays a role in leukocyte homing to inflamed tissues and in the perpetuation of inflammation, and therefore, tissue damage.

Indeed, joint inflammation was alleviated in *Cxcl10*^−/−^ mice (Figure 1 and Figure 3), and this was accompanied by a significant reduction in macrophages, which constituted the largest immune cell population in joints following infection (Figure 4B) [38,42,43]. These activated macrophages could be a main cellular reservoir for CHIKV persistence during the late stages of infection [38,42] and contribute to sustained inflammation. In addition to recruiting immune cells, CXCL10 signaling could directly stimulate viral replication, for instance, human immunodeficiency virus 1 (HIV-1) replication in macrophages and lymphocytes [44]. In this study, we unexpectedly observed a reduction in CHIKV/ONNV in *Cxcl10*^−/−^ compared to WT mouse feet at the late stages (days 4/6 and thereafter, respectively) (Figure 1B and Figure 3B), suggesting a pro-viral role for CXCL10 signaling. However, the absence of CXCL10 did not impact viremia (Figure 1A and Figure 3A), suggesting that CXCL10 signaling is dispensable for systemic viral dissemination. Therefore, a reduction of viral loads in *Cxcl10*^−/−^ mouse feet could be due to fewer macrophages that are supportive of viral replication in *Cxcl10*^−/−^ than WT feet [38,42].

Statins are clinically approved, cholesterol-lowering drugs. They may also have an anti-inflammatory feature by potently inhibiting CXCL10. A recent meta-analysis of rheumatoid arthritis disease severity in patients treated with statins or a placebo revealed that statins may be effective in improving rheumatoid arthritis disease activity measured by the disease activity score, tender joint count, and swollen joint count [45]. We also demonstrated that the inhibition of CXCL10 by statins could alleviate CHIKV arthritis (Figure 2). Moreover, statins can directly interfere with viral replication, such as Ebola [46], Zika [47], and influenza [48] virus. Therefore, statins could represent a potential drug for the treatment of CHIKV arthritis. However, statins could also interfere with antiviral immune responses due to their inhibition on STAT1 [49,50], RELA [51], and the NF-κB complex [49,52]. Therefore, clinical treatment regimens, including the dose, frequency, and timing of administration, need to be optimized to minimize side effects.

Intriguingly, the viral RNA loads in macrophages and neutrophils of *Cxcl10*^−/−^ mouse feet were ~3–6-fold lower than those of WT mice (Figure 5). Macrophages and neutrophils were the predominant immune cell types in mouse feet following alphaviral infection (Figure 4). As such, it is plausible that CXCL10 signaling could directly promote alphaviral replication in macrophages and/or other immune cells. CXCL10 is also a chemoattractant for CD4^+^ T cells, which, though constituting only a small fraction of immune infiltrates during the second peak of foot swelling, are believed to underlie CHIKV-induced inflammation in mice [16,28,53]. However, CD4^+^ T cells were recruited to footpads by day 6 following ONNV infection and the numbers of CD4^+^ T cells in *Cxcl10*^−/−^ feet were no different than those in WT feet (data not shown).

In summary, our results demonstrate that CXCL10 signaling contributes to alphaviral replication and the pathogenesis of alphavirus-induced arthritis. Future work is required to elucidate how CXCL10 signaling facilitates alphaviral replication.
